# Calla Lilies

**Published:** 1875-06

**Authors:** 


Calla Lilies.—Now is the time to make preparations for a splendid supply of this magnificent flower all through the winter. In June you must take your callas out of doors, and turn the pots containing them over on their sides under a tree or in some shady place, and there leave them through the hot Summer months, giving them no attention whatever. Of course the old leaves die and drop off, and the earth in the pots bakes into the consistency of brick. One would think such harsh treatment would be the death of a flower, but, on the contrary, the calla likes it. In September, bring the pot in, and begin to give the plants water. A very short time suffices to start them into growth. As soon as the leaves appear, make the water quite warm. The result will be that you will have splendid flowers, and your callas will never be without flowers through the Winter; often as many as four or five will be open on a single plant at once, and never remove the new ones which form about the old plant, but, as they grow, shift the plants into larger pots.

---

Why is it that the beauty of many females fades so soon? One special cause is the neglecting of air and exercise. Daily out-door employment is the boon of life. Weakness, lassitude, indigestion, nervous affections, headache, loss of appetite, liability to sudden colds, and a fading complexion, as inevitably follow indolence and confinement as the wilting of a plant results from the deprivation of light.

---

Bounty, being free itself, thinks all others so.

